# Effect of Overheating on the Tensile Properties of Nickel-Based Superalloy GH4720Li

**DOI:** 10.3390/ma17102351

**Published:** 2024-05-15

**Authors:** Anqi Wang, Zhicheng Liu, Ruoyao Cui, Yangyang Wu, Di Zhang, Xiaogang Wang

**Affiliations:** 1 Key Laboratory of Advanced Design and Simulation Techniques for Special Equipment, Ministry of Education, College of Mechanical and Vehicle Engineering, Hunan University, Changsha 410082, China; wang161028@hnu.edu.cn (A.W.); zhruoyao2021@hnu.edu.cn (R.C.); xgwang@hnu.edu.cn (X.W.); 2 Hunan Aviation Powerplant Research Institute, Aero Engine Corporation of China, Zhuzhou 412002, China; wuyangyang1005@sina.cn (Y.W.); zhangdi0524@sina.cn (D.Z.)

**Keywords:** superalloy, one engine inoperative, tensile properties, overheating, yield strength

## Abstract

Aero-engines can be exposed to One Engine Inoperative (OEI) conditions during service, and the resulting overheating effect may significantly impact their structural integrity and flight safety. This paper focuses on the influence of overheating on the microstructural evolution and tensile properties of the GH4720Li alloy, a nickel-based polycrystalline superalloy commonly used in turbine disks. Based on the typical OEI operating conditions of a real aero-engine, a series of non-isothermal high-temperature tensile tests involving an OEI stage of 800 °C were conducted. The effects of OEI-induced overheating on the microstructure and tensile properties of the GH4720Li alloy were investigated. The results showed that, after OEI treatment, the primary γ′ phase in this alloy was partially dissolved. The GH4720Li superalloy also exhibited numerous microcracks at the grain boundaries, resulting in complex effects on its tensile properties. The alloy’s yield strength and ultimate tensile strength were slightly decreased, whereas its ductility decreased considerably. The OEI-induced embrittlement phenomenon was mainly caused by the non-uniform distribution of the tertiary γ′ phase within grains. The formation of microcracks nucleated at the interfaces between the primary γ′ precipitates and γ matrix phase was another key factor.

## 1. Introduction

The GH4720Li alloy is a Ni-Cr-Co precipitation-strengthened nickel-based high-temperature alloy (a Chinese alloy grade similar to Udimet 720Li). It is mainly used in the critical rotating parts of turbine engines operating below 800 °C, such as turbine disks and blades. Harmful carbide formation is limited by reducing certain interstitial elements (such as C and B) in the alloy’s chemical composition, improving its mechanical properties [1]. The alloy is prepared using the Vacuum Induction Melting (VIM)-Electro-slag Remelting (ESR)-Vacuum Arc Remelting (VAR) triple-melting process. Current research [2] shows that the VIM process can effectively reduce the number and size of alloy inclusions. Added elements such as Al-Ti-Nb to the GH4720Li alloy increase the γ′ phase content, so that the high-temperature strength, fatigue resistance, creep resistance, and corrosion resistance improve [3]. In recent years, scholars have conducted extensive studies on the tensile and fatigue properties of the GH4720Li alloy [4,5]. Among them, Jackson et al. [6] studied the microstructural evolution of the Udimet 720Li alloy’s forging structure during heat treatment. They divided the precipitated phases of different sizes and distributions into the primary γ′ phase, secondary γ′ phase, and tertiary γ′ phase. Chen et al. [7] explored the strengthening mechanism of the γ′ phase during the thermal deformation of the Udimet 720Li alloy. They found a so-called ‘pinning effect’ during the thermal deformation of the primary γ′ phase, which suppressed γ matrix sliding. At the same time, grain size was also one of the key parameters affecting the deformation mechanism of the alloy. Nevertheless, existing works on the GH4720Li alloy lack sufficient research on the impact of overheating on its tensile properties. There is also a lack of relevant research on the effects of overheating on the microstructural evolution and tensile properties of the GH4720Li superalloy.

As a critical component in advanced aero-engines, turbine discs are manufactured from nickel-based superalloys. Nickel-based superalloys generally have excellent high-temperature mechanical properties and oxidation resistance [8]. Under overheating conditions, important microstructure characteristics of nickel-based polycrystalline superalloys, such as their grain size, grain morphology, volume fraction, and the distribution of their precipitated γ′ phase will change, leading to potential changes in superalloys’ mechanical properties [9]. In recent years, research on the effects of overheating on the microstructure and mechanical properties of nickel-based alloys has mainly focused on the alloys’ overheating and subcooling rates. Among them, Utada and Sasaki [10] found that the heating and cooling rates of overheating treatment significantly impact the microstructure and mechanical properties of the nickel-based superalloy Waspaloy. By setting a reasonable heat treatment temperature and duration, the alloy’s mechanical properties can be significantly improved. While studying the tensile properties of the Udimet 720Li alloy, Monajati [11] found that the grain size and deformation mechanism of the primary γ′ phase contribute significantly to the yield strength of the alloy, while the grain size and primary γ′ highly depend on temperature.

However, aero-engines’ key components are exposed to harsh environments with complex loading conditions when serviced [12]. When an aircraft is in the air, one engine may fail to operate due to unpredictable reasons, such as One Engine Inoperative (OEI) conditions [13]. When an aircraft encounters an OEI situation, the remaining engine must be subjected to a short-term high-power emergency state to ensure flight safety. In this situation, the operating temperature of key engine components will rise sharply, causing them to overheat [14]. The overheating process is often accompanied by changes in the material’s microstructure and mechanical properties, which may affect the structural strength and service safety of turbine components in aero-engines. Therefore, studying the effects of overheating on the tensile properties of high-temperature alloys under OEI conditions is important for ensuring aerospace engines’ operational safety.

This paper focuses on the effects of OEI overheating on the tensile properties of the GH4720Li alloy and its potential deformation mechanism. First, the OEI process inserts an overheating treatment (similar to a heat treatment under loading conditions) at base temperatures and stress levels corresponding to the normal service conditions of aero-engines. Secondly, the temperature level and number of treatments were considered to be two key factors of OEI working conditions. Lastly, by further analyzing the tensile properties and microstructure of alloys treated with different OEI states, the microscopic influencing mechanism of the tensile property transformation of the materials is revealed.

## 2. Materials and Methods

### 2.1. Material

**Figure 1:**
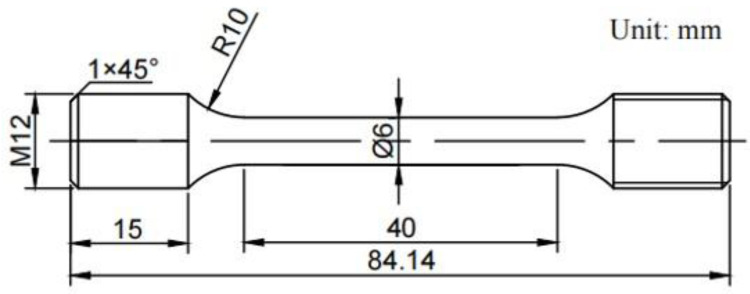
Geometric dimensions of the tensile specimen.

The material studied was GH4720Li, a nickel-based polycrystalline superalloy. This alloy was prepared following a standard sub-solid solution treatment (1110 °C-4 h-oil cooling) and an aging treatment (650 °C-24 h-air cooling + 760 °C-16 h-air cooling). Table 1 shows the main chemical composition of the GH4720Li alloy. The tensile specimen was cut in the radial direction from the turbine disk ingot blank. Figure 1 shows the geometric dimensions of the specimen.

**Figure 2:**
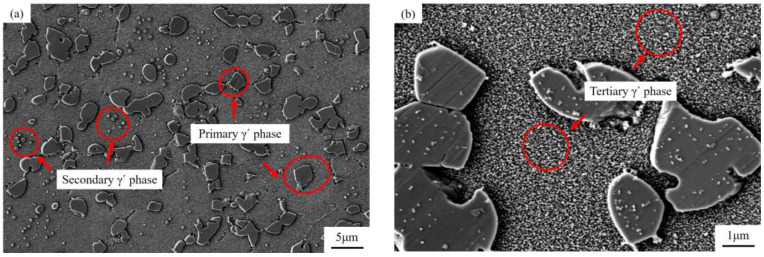
SEM images showing the microstructure of the GH4720Li alloy: the (**a**) primary γ′ phase and the secondary γ′ phase; the (**b**) tertiary γ′ phase.

Scanning electron microscopy (SEM) was used to characterize the microstructure of the GH4720Li alloy. The samples were electrolytically polished using a solution of 150 mL of H_3_PO_4_ + 10 mL of H_2_SO_4_ + 15 g of CrO_3_. The representative SEM images shown in Figure 2 show that the strengthening phases of the GH4720Li alloy mainly comprise three different γ′ phase types, namely primary γ′, secondary γ′, and tertiary γ′. The primary γ′ phase is located at the edge of grain boundaries, with a diameter of 1~10 μm. The secondary γ′ phase is mainly distributed inside the grains, with a diameter of 70~120 nm. The tertiary γ′ phase, which has very fine particles (15~50 nm in diameter), is embedded in the γ matrix in a rather dispersed manner.

Based on the results of microstructural evolution during cooling after the solidification of the Udimet 720Li alloy [15], it is known that the temperature at which the γ′ phase is completely dissolved is 1160 °C. The superalloy undergoes sub-solid solution treatment at 1100 °C to dissolve part of the γ′ phase. The remaining part of the γ′ phase is retained at the grain boundaries, which is the primary γ′. Its ‘pinning effect’ improves the strength of the material, representing the primary strengthening mechanism of the alloy. Moreover, the presence of the secondary γ′ and tertiary γ′ also improves the alloy’s tensile strength. However, it remains an open question whether these microstructural features can sustain OEI treatment and how overheating affects the alloy’s strengthening mechanism. In particular, microcrack generation at grain boundaries is an important issue in this work. Previous studies [16] showed that oxidation-assisted grain boundary embrittlement and microcrack generation can significantly impact the tensile properties of alloys.

### 2.2. Experimental Methods

**Figure 3:**
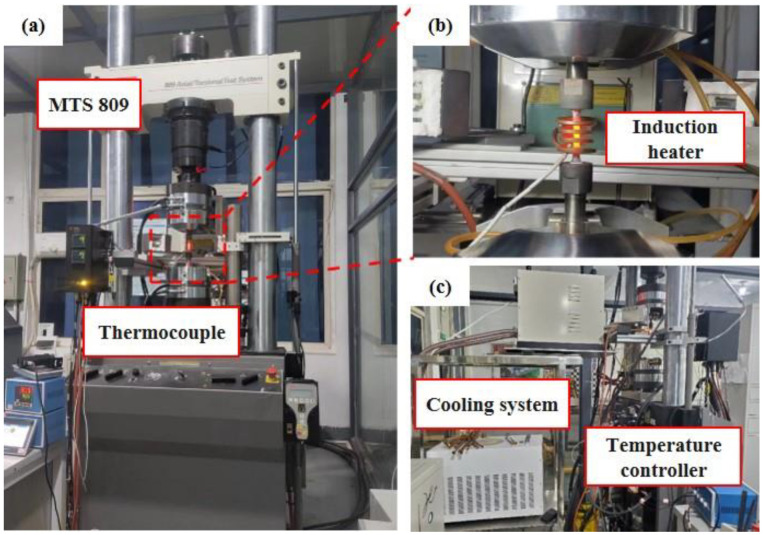
Experimental setup of the thermo-mechanical testing system: (**a**) a global view of the system; (**b**) the induction heater; (**c**) the cooling system and temperature controller.

Figure 3 shows the experimental setup for the thermo-mechanical testing system. The experimental procedure was as follows:

Firstly, a series of high-temperature tensile tests (with temperature levels of 400 °C, 600 °C, 700 °C, and 800 °C) were conducted in accordance with ASTM E8/E8M standards using an MTS Model 809 hydraulic testing machine to evaluate the alloy’s tensile properties including its yield strength *σ_Y_*, ultimate tensile strength *σ_UTS_*, elongation to failure ε*_f_*, etc. The tensile tests were conducted at a constant strain rate of 1 × 10^−3^ s^−1^. The heating rate of the furnace used was 3 °C/s, and the holding time before tensile testing was 20 min. During tensile testing, the temperature was measured in real-time using a thermocouple. The temperature deviation was controlled within ±2 °C.

Secondly, specimens were processed using an OEI treatment and the tensile properties of these OEI-treated samples were re-evaluated through the same tensile testing procedure as before. The OEI treatment involved an induction heater (Figure 3b) enabling rapid heating rates as high as 60 °C s^−1^ to simulate real OEI conditions. Multiple OEI conditions were performed by subjecting the specimen to OEI conditions once and cooling it to room temperature before proceeding to the next.

Finally, the microstructure and tensile fractography of the alloy after OEI treatment are characterized to analyze the effect of microstructural changes on the tensile properties of the alloy.

**Figure 4:**
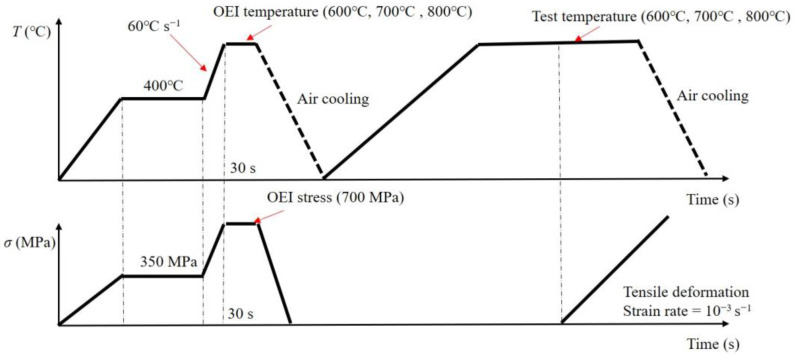
Schematic diagram of high-temperature tensile test conditions after an OEI treatment.

In this work, OEI conditions were determined based on the actual OEI conditions of a specific type of aero-engine. Figure 4 shows the high-temperature tensile test conditions after OEI treatment. Firstly, before an OEI treatment occurred, the basic temperature level was set to 400 °C, and the basic stress level was set to 350 MPa, which was consistent with the engine’s normal operating conditions. When an OEI treatment occurred, the temperature was raised rapidly to an ultra-high heating rate of 60 °C/s. Three different levels of OEI temperatures (600 °C, 700 °C, and 800 °C) were chosen to represent OEI severity. Meanwhile, the applied stress to the specimen was simultaneously increased to a higher level of 700 MPa. The number of OEI treatments was another important variable in this work, and three different levels were adopted (1, 2, and 8). The detailed information is reported in Table 2.

The OEI treatment’s effect on the alloy’s tensile properties was evaluated by performing tensile tests on OEI-treated specimens. The alloy’s relevant mechanical properties were then analyzed and compared to samples without OEI treatments. The temperature levels adopted for ordinary tensile testing were the same as the OEI temperature levels, as shown in Table 2.

## 3. Results and Analysis

### 3.1. High-Temperature Tensile Test Results

**Figure 5:**
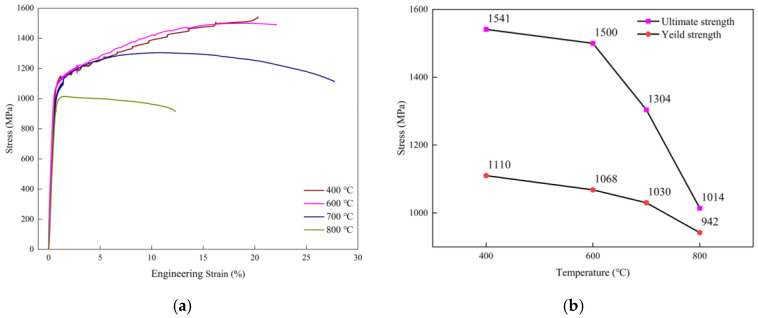
(**a**) Stress–strain curves of the GH4720Li alloy at 400 °C, 600 °C, 700 °C, and 800 °C. (**b**) The yield strength and ultimate tensile strength obtained by the alloy at each test temperature.

**Figure 6:**
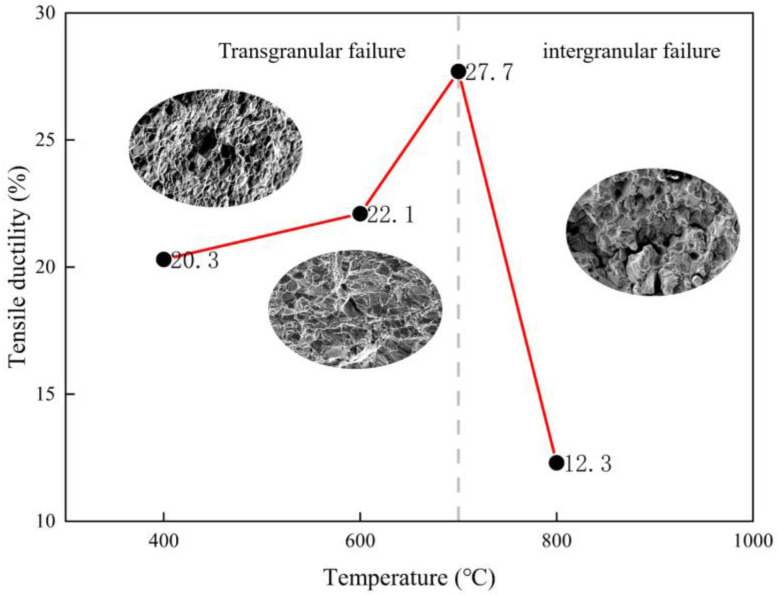
Plot of the GH4720Li alloy’s tensile ductility versus temperature.

The GH4720Li alloy underwent high-temperature tensile tests at 400 °C, 600 °C, 700 °C, and 800 °C. The engineering stress–engineering strain curve of the material is shown in Figure 5a. In Figure 5b, the yield strength *σ_Y_* and ultimate tensile strength *σ_UTS_* at 600 °C are relatively close to those at 400 °C, whereas *σ_Y_* decreases significantly at 700 °C and 800 °C. The strength of alloy GH4720Li is temperature independent up to 400 °C and 600 °C, which agrees with conventional tensile test results [17,18]. Above 700 °C, its strength is highly temperature dependent and decreases rapidly, suggesting a change in the deformation mechanism. The three temperature regimes have markedly different tensile ductility. Firstly, at low temperatures below 600 °C, the GH4720Li superalloy remains ductile with a failure strain exceeding 20%. Secondly, a significant increase in tensile ductility is observed at intermediate temperatures in the range of 700 °C, with a maximum failure strain of 27.7%. Thirdly, ductility decreases significantly at high temperatures of 800 °C. Notably, the alloy’s tensile ductility at 800 °C is less than half that at 700 °C. The dramatic loss in ductility at 800 °C is consistent with observations from other superalloy systems [16,19,20,21]. One reason for this is that below 800 °C, transgranular damage is the main failure mode of the alloy. Above 800 °C, a transition occurs between ductile transgranular and quasi-brittle intergranular failure. Therefore, intergranular crack propagation in this regime limits tensile ductility, as shown in Figure 6, where tensile ductility is plotted against temperature.

### 3.2. High-Temperature Tensile Test Results after OEI-State Treatment

**Figure 7:**
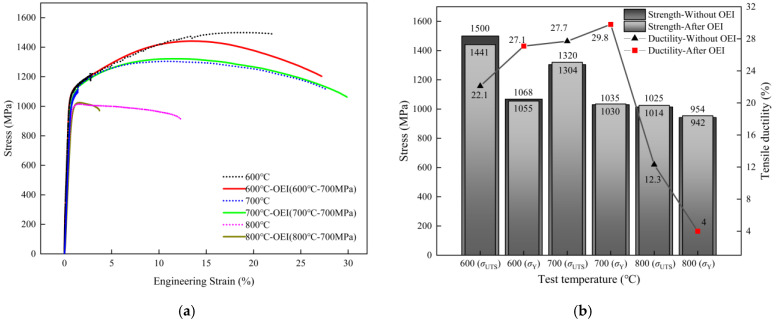
(**a**) The high-temperature tensile stress–strain curve of the GH4720Li material after OEI-state treatment. (**b**) The material’s tensile property data such as yield strength, ultimate strength, and elongation at break.

Figure 7 a shows the stress–strain curves of the GH4720Li alloy with and without OEI treatment. Comparisons between specific test conditions and material tensile property data such as its yield strength, ultimate strength, and elongation at break are listed in Figure 7b (changes in tensile properties before and after OEI-state treatment). The results show that under an OEI test stress of 700 MPa and temperature levels of 800 °C, 700 °C, and 600 °C, changes in yield strength (0.2% plastic strain), ultimate tensile strength, and elongation at break are not monotonic. It is worth noting that after the OEI-state treatment at 800 °C-700 MPa-30 s, tensile ductility decreased by 67.5%, whereas yield strength and ultimate tensile strength increased by 1%. After the OEI-state treatment at 700 °C-700 MPa-30 s, the alloy’s tensile properties showed almost no change, and only ductility increased by 7.6%. After the lower OEI-state treatment at 600 °C-700 MPa-30 s, the ultimate tensile strength of the alloy decreased by 4%, whereas the yield strength increased by 2.1%, and the tensile plasticity increased by 22.6%. Based on the evaluation results above, after the strict OEI treatment at 800 °C-700 MPa-30 s, significant changes in elongation at break seriously affect the safety of the engine turbine’s components. Therefore, this OEI treatment condition was selected as the working condition for multi-OEI-state processing.

**Figure 8:**
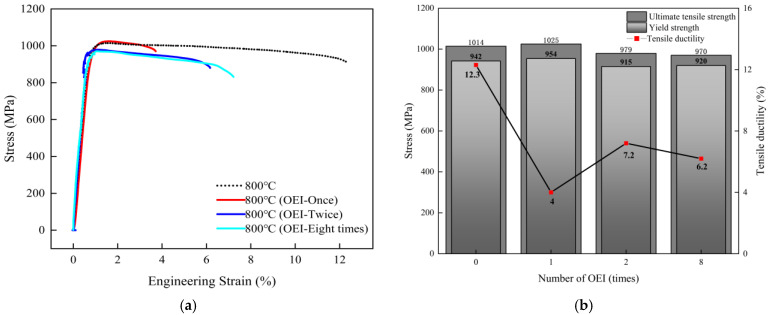
(**a**) The 800 °C high-temperature tensile stress–strain curve of the GH4720Li material after multiple OEI state treatments. (**b**) The material’s tensile property data, such as yield strength, ultimate strength, and elongation at break, after multiple OEI state treatments.

As shown in Figure 7, the OEI-state treatment at 800 °C-700 MPa-30 s has a greater impact on the mechanical properties of GH4720Li in a one-time overheating-state treatment situation. To explore the impact of multiple overheating-state treatments on the GH4720Li alloy, the overheating state at 800 °C-700 MPa-30 s was selected as the typical working condition of one-time overheating treatments. Figure 8 a shows the high-temperature tensile stress–strain curves of the GH4720Li alloy after different OEI treatment times. The results show that, after two and eight OEI treatments, the ultimate tensile strength, yield strength, and fracture elongation decreased to a certain extent. Compared to the tensile properties at 800 °C without OEI treatment, the yield strength of the GH4720Li superalloy after two and eight overheating treatments decreased by 27 MPa and 22 MPa, the ultimate strength decreased by 35 MPa and 44 MPa, and the ductility declined by 41% and 50%, respectively. Specific test data are shown in Figure 8b.

### 3.3. Effect of OEI-State Treatment on Precipitate Morphology

**Figure 9:**
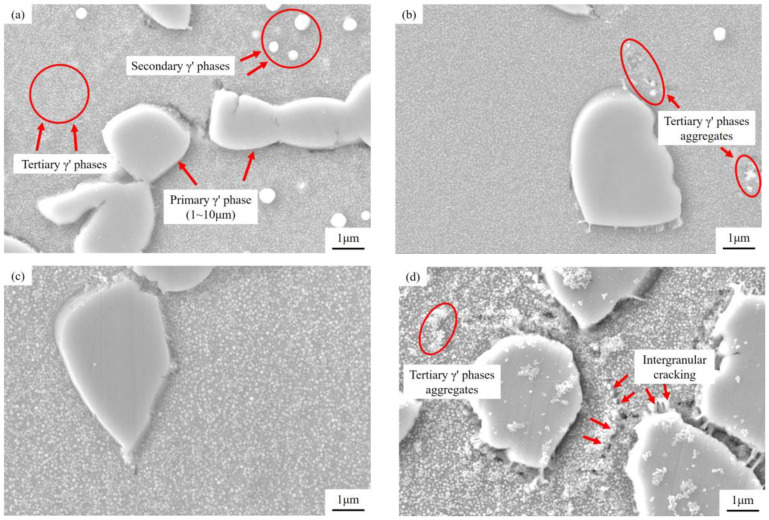
The microstructure of the GH4720Li alloy after OEI treatment: (**a**) without OEI treatment; (**b**) after OEI treatment at 600 °C-700 MPa-30 s; (**c**) after OEI treatment at 700 °C-700 MPa-30 s; (**d**) after OEI treatment at 800 °C-700 MPa-30 s.

To understand the effects of OEI treatment on the GH4720Li alloy’s microstructure, SEM images with and without OEI treatment are shown in Figure 9. In Figure 9a, the distribution of the tertiary γ′ phase in the alloy’s microstructure without OEI treatment is relatively uniform, with no apparent cracks at the grain boundaries. At the same time, there is a small amount of the secondary γ′ phase distributed around the primary γ′ phase. Figure 9b shows the microstructure of the alloy after the OEI state treatment at 600 °C-700 MPa-30 s. Only a small amount of the tertiary γ′ phase gathers at the grain boundaries, and the alloy microstructure distribution is relatively uniform. Figure 9c shows the microstructure of the alloy after the OEI-state treatment at 700 °C-700 Mpa-30 s, and the secondary γ′ phase is completely dissolved. Figure 9d shows that after the OEI-state treatment at 800 °C-700 Mpa-30 s, obvious microcracks appear at the alloy grain boundaries. The tertiary γ′ phase is aggregated and unevenly distributed around the γ matrix phase of the alloy.

**Figure 10:**
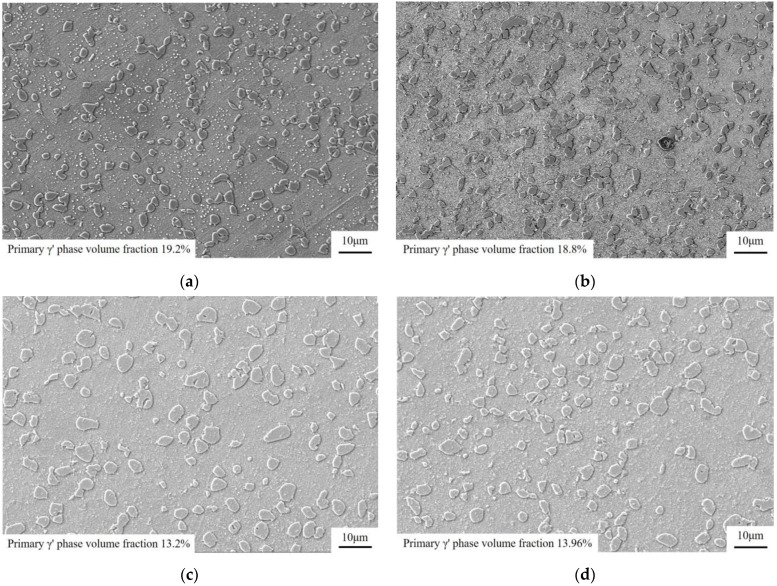
(**a**) The volume fraction of the primary γ′ phase in the initial alloy. (**b**) The volume fraction of the primary γ′ phase after constant temperature and load treatment at 400 °C for 5 min. (**c**) The volume fraction of the primary γ′ phase after OEI treatment at 800 °C-700 MPa-30 s. (**d**) The volume fraction of the primary γ′ phase after OEI treatment at 800 °C-700 MPa-30 s with additional thermal insulation at 800 °C for 20 min.

In this study, the sample’s microstructure changed after OEI-state treatment. Figure 10b shows that overheating the material at 800 °C for 30 s causes the volume fraction of the primary γ′ phase to rapidly decrease to the content levels at 800 °C. At the same time, the dissolution of the second γ′ phase is promoted during this process. A large amount of the tertiary γ′ phase also accumulates between the grains, creating grain boundary voids in the alloy. Cavitation at grain boundaries (Figure 9d) can also decrease tensile ductility, as reported by Andersson [22]. According to Jackson [6], the second γ′ phase content relates to the cooling rate, and the size and distribution of the tertiary γ′ phase are related to the treatment’s temperature. However, the concentration of intergranular microcracks in the tertiary γ′ phase and void generation may be caused by rapid temperature increases during the OEI-state treatment. When the material is rapidly heated from low temperatures of 400 °C to 800 °C, the precipitated phase in the alloy is thermally activated. As the temperature increases, the primary γ′ phase rapidly dissolves and the volume fraction decreases. Additionally, the secondary γ′ phase is completely dissolved, and the tertiary γ′ phase cannot flow through the grain boundaries due to the obstruction caused by accumulation. Stress concentration occurs, leading to grain boundary hole formation. The strength of such high-temperature alloys mainly depends on the size and distribution of the primary γ′ phase [23,24]. Therefore, after being treated in the OEI state and air-cooled to room temperature, the tensile strength of the sample has almost no change in subsequent high-temperature tensile tests at 800 °C. However, the tensile plasticity of the material decreases significantly. Satoshi’s research [10] on the Waspaloy alloy proves this point. The tensile properties of the GH4720Li alloy did not show significant differences from alloys subjected to 700 °C OEI conditions. However, after experiencing an OEI temperature of 600 °C, the ultimate tensile strength of the material decreased slightly, which may be related to material errors.

OEI-state treatment significantly impacts the primary γ′ volume fraction of the GH4720Li alloy and causes irreversible damage to its microstructure. The influence of an OEI treatment on the tensile properties of the GH4720Li alloy was determined by analyzing the volume fraction of the primary γ′ phase during the short-term overheating treatment of the alloy at 800 °C. Statistical results are shown in Figure 10.

Figure 10 shows that, when the GH4720Li alloy is kept at 400 °C for 5 min, the volume fraction of the primary γ′ phase shows almost no change. Subsequently, the alloy was subjected to an OEI-state treatment at 800 °C-700 MPa-30 s, and the volume fraction of the alloy’s primary γ′ phase quickly dropped to 13.2%. After passing the OEI conditions, the alloy was subjected to a high-temperature tensile test at 800 °C. During the process of heating the material to 800 °C for 20 min, the volume fraction of the primary γ′ phase in the alloy was restored to a certain extent due to the thermal activation energy.

**Figure 11:**
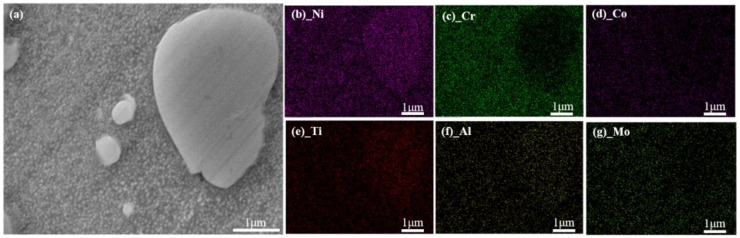
The GH4720Li alloy’s element distribution before OEI treatment (heated to 400 °C and maintained for 5 min). (**a**) SEM image of the alloy. Distribution of different elements in the alloy: (**b**) Ni. (**c**) Cr. (**d**) Co. (**e**) Ti. (**f**) Al. (**g**) Mo.

**Figure 12:**
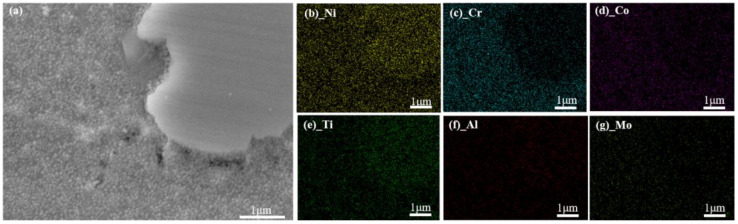
The GH4720Li alloy’s element distribution after OEI treatment (heated to 800 °C and maintained for 30 s). (**a**) SEM image of the alloy. Distribution of different elements in the alloy: (**b**) Ni. (**c**) Cr. (**d**) Co. (**e**) Ti. (**f**) Al. (**g**) Mo.

**Figure 13:**
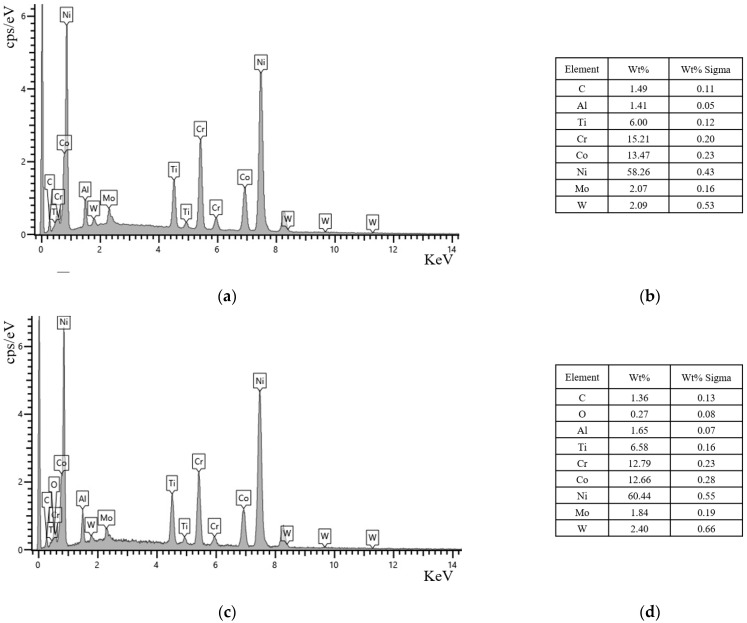
(**a**) The GH4720Li alloy’s energy spectrum (before OEI treatment); (**b**) the GH4720Li alloy’s element content table (before OEI treatment); (**c**) the GH4720Li alloy’s energy spectrum (after OEI treatment); (**d**) The GH4720Li alloy’s element content table (after OEI treatment).

Energy Dispersive Spectrometer (EDS) analysis shows the GH4720Li alloy’s chemical element changes before and after OEI-state treatment (as shown in Figure 11 and Figure 12). The changes in the chemical element content of GH4720Li alloy before and after OEI treatment are shown in Figure 13. Research shows that, before the GH4720Li alloy undergoes OEI-state treatment (heated to 400 °C for 5 min), the element distribution within the alloy is relatively obvious, as shown in Figure 11. The C element content is only 1.49%, and other elements in the alloy are close to the original alloy contents. After the GH4720Li alloy underwent OEI-state treatment (rapidly heated to 800 °C for 30 s), the element distribution in the alloy did not change significantly, as shown in Figure 12. The presence of the O element proves that the alloy was slightly oxidized. The above analysis shows that, after the GH4720Li alloy was treated in this OEI state, the overall element distribution of the alloy changed slightly. However, the volume fraction of the strengthening phase changed significantly. The tertiary γ′ phase was also freed at the edge of the alloy grain boundary.

**Figure 14:**
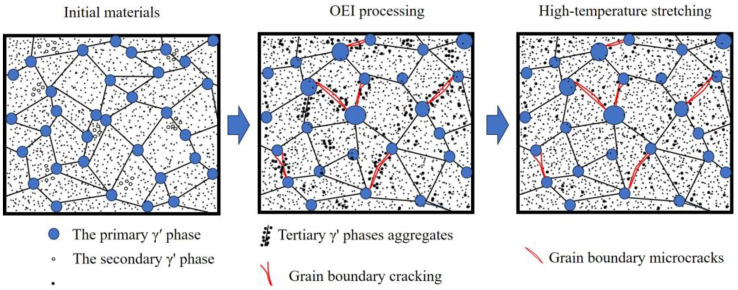
OEI (800 °C-700 MPa-30 s) status processing schematic diagram.

Changes in the microstructure of GH4720Li after exposure to different OEI treatment temperatures, as shown in Figure 13, reveal the influencing mechanism of the OEI state. The material’s microstructural changes after the OEI-state treatment at 800 °C-700 MPa-30 s are the most significant. During this process, the alloy produced many microcracks and accumulated a large amount of the tertiary γ′ phase at the grain boundaries. Due to the subsequent heating of the material to 800 °C for 20 min, the damage caused by the treatment decreased, resulting in little change in alloy strength but numerous microcracks that significantly reduced tensile elongation. Figure 14 shows the schematic diagram of its impact.

**Figure 15:**
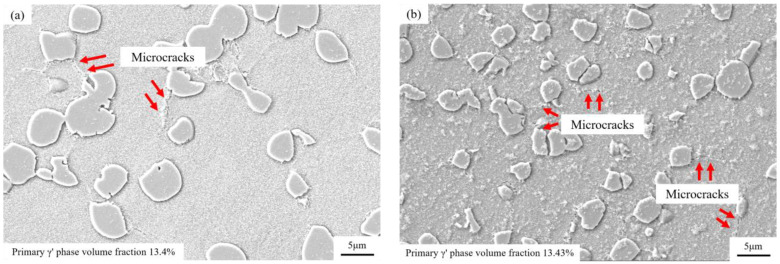

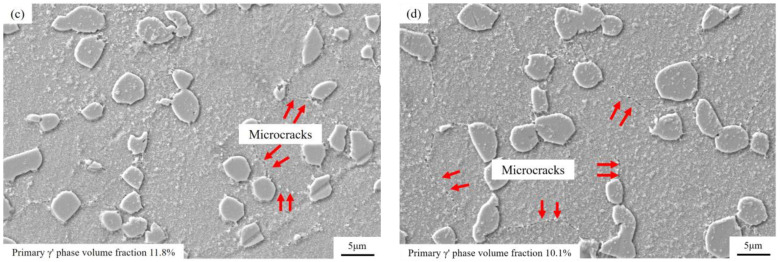
The microstructure of the GH4720Li alloy after multiple overheating state treatments: (**a**) without an OEI-state treatment at 800 °C; (**b**) after one OEI-state treatment; (**c**) after two OEI-state treatments; (**d**) after eight OEI-state treatments and status processing.

After the OEI process at 800 °C-700 MPa-30 s, the alloy suffers irreversible damage, such as intergranular microcracks that significantly affect its tensile elongation. Therefore, this OEI working condition was used as a single processing condition to explore the influence of OEI-state times on the alloy’s tensile properties. SEM was also used to observe the microstructural changes of the GH4720Li alloy after undergoing zero, one, two, and eight OEI-state treatments. Figure 15 shows the observation results, where the volume fractions of the primary γ′ phase are 13.4%, 13.43%, 11.8%, and 10.1% in the GH4720Li alloys that underwent zero, one, two, and eight OEI-state treatments (statistical method reference standard [25]), respectively. Statistics were calculated based on the average grain size of the primary γ′ phase in Figure 15. The results are shown in Table 3. OEI treatment rapidly reduces the volume fraction of the primary γ′ phase in the alloy to content levels at 800 °C while the primary γ′ phase dissolves and thickens. Research [26] shows that an increase in average grain size will reduce material grain boundaries, which, in turn, weakens grain boundary strength.

Therefore, different OEI treatments at 800 °C-700 MPa-30 s significantly influence alloy microstructure. For instance, as treatment times increase, the volume fraction of the primary γ′ phase decreases and the average grain size increases, reducing the alloy’s ultimate tensile strength and yield strength. Additionally, microcracks between alloys lead to a significant reduction in the alloy’s elongation at break.

The changes in the microstructure of the GH4720Li alloy after the above OEI treatment can be summarized as follows: Firstly, OEI treatment led to the partial dissolution of the primary γ′ phase within the GH4720Li alloy. This phase is critical to the alloy’s strength and usually exists around the gamma phase. Secondly, microcracks were observed at grain boundaries following OEI treatment. These microcracks are detrimental to the material’s integrity and contribute to embrittlement. Thirdly, the embrittlement phenomenon was primarily attributed to the non-uniform distribution of the tertiary γ′ phase within the grains. This uneven distribution causes stress in concentration areas, further contributing to microcrack formation and growth. Finally, overheating at 800 °C for 30 s notably reduced the volume fraction of the primary γ′ phase to levels typical of 800 °C. It also promoted the dissolution of the secondary γ′ phase and led to an accumulation of the tertiary γ′ phase between grains, creating grain boundary voids.

## 4. Conclusions

An OEI state’s effect on the tensile properties of GH4720Li was studied using a thermo-mechanical testing system that could perform complex thermal history tests. The main conclusions derived from this work are as follows:

1. After the GH4720Li alloy was overheated at different temperatures for 30 s and cooled to room temperature in air, its tensile properties changed dramatically after OEI treatment at 800 °C, with its tensile ductility reduced by more than 50%. Lowering the overheating temperature (700 °C) had little effect on the tensile properties of the material.
2. The GH4720Li alloy’s microstructure changed significantly after OEI treatment. In the most stringent OEI treatment, the primary γ′ phase of the alloy rapidly dissolved. The accumulation of the tertiary γ′ phase led to numerous irreversible microcracks between the grains. These damage mechanisms significantly decreased the elongation at break of the alloy after OEI treatment, and multiple OEI treatments exacerbated this effect.
3. As the GH4720Li alloy was subjected to multiple OEI treatments at 800 °C-700 MPa-30 s, the volume fraction of the primary γ′ phase continued to decrease, the average size continued to increase, and, at the same time, irreversible microcrack damage occurred between the grain boundaries. As a result, the ultimate tensile strength and yield strength of the GH4720Li alloy decreased, as did its tensile plasticity.

## Figures and Tables

**Table 1:** Nominal chemical composition of the GH4720Li alloy (mass fraction/%).

Cr	Co	W	Mo	Al	Ti	C	B	Zr	Ni
16	14.7	1.25	3	2.5	5	0.015	0.015	0.05	margin

**Table 2:** The OEI conditions adopted in this work.

OEI Temperature	OEI Stress	Duration of Action	Number of OEI Stage	Heating Rate to the OEI Stage
800 °C	700 MPa	30 s	1	60 °C s^−1^
800 °C			2	
800 °C			8	
700 °C			1	
600 °C			1	

**Table 3:** The microstructure of the GH4720Li alloy after multiple overheating treatments.

OEI-Times	0	1	2	8
Primary γ′ volume fraction *f*/%	13.4	13.43	11.8	10.1
Primary γ′ grain size *d*/μm	4.3	4.58	4.451	4.78

## Data Availability

Dataset available on request from the authors.
